# The short-term safety and efficacy of fluoxetine in depressed adolescents with alcohol and cannabis use disorders: a pilot randomized placebo-controlled trial

**DOI:** 10.1186/1753-2000-3-11

**Published:** 2009-03-19

**Authors:** Robert L Findling, Maria E Pagano, Nora K McNamara, Robert J Stansbrey, Jon E Faber, Jacqui Lingler, Christine A Demeter, Denise Bedoya, Michael D Reed

**Affiliations:** 1 Department of Psychiatry, University Hospitals Case Medical Center/Case Western Reserve University, Cleveland, Ohio, USA; 2 Department of Pediatrics, Akron Children's Hospital, Akron, Ohio, USA

## Abstract

**Background:**

The objective of this study was to examine whether fluoxetine was superior to placebo in the acute amelioration of depressive symptomatology in adolescents with depressive illness and a comorbid substance use disorder.

**Methods:**

Eligible subjects ages 12–17 years with either a current major depressive disorder (MDD) or a depressive disorder that were also suffering from a comorbid substance-related disorder were randomized to receive either fluoxetine or placebo in this single site, 8-week double-blind, placebo-controlled study. The primary outcome analysis was a random effects mixed model for repeated measurements of Children's Depression Rating Scale-Revised (CDRS-R) scores compared between treatment groups across time.

**Results:**

An interim analysis was performed after 34 patients were randomized. Based on the results of a futility analysis, study enrollment was halted. Twenty-nine males and 5 females were randomized to receive fluoxetine (n = 18) or placebo (n = 16). Their mean age was 16.5 (1.1) years. Overall, patients who received fluoxetine and placebo had a reduction in CDRS-R scores. However, there was no significant difference in mean change in CDRS-R total score in those subjects treated with fluoxetine and those who received placebo (treatment difference = 0.19, S.E. = 0.58, F = 0.14, p = .74). Furthermore, there was not a significant difference in rates of positive urine drug toxicology results between treatment groups at any post-randomization visit (F = 0.22, df = 1, p = 0.65). The main limitation of this study is its modest sample size and resulting low statistical power. Other significant limitations to this study include, but are not limited to, the brevity of the trial, high placebo response rate, limited dose range of fluoxetine, and the inclusion of youth who met criteria for depressive disorders other than MDD.

**Conclusion:**

Fluoxetine was not superior to placebo in alleviating depressive symptoms or in decreasing rates of positive drug screens in the acute treatment of adolescents with depression and a concomitant substance use disorder.

## Background

Depression is a common condition amongst teenagers, with prevalence estimates of approximately 3–5% [1,2]. Depression during adolescence is a serious illness that is associated with impairments in functioning, school performance, personal relationships, and suicidal behavior [3,4]. In addition, an estimated 20%–30% of adolescents with major depressive disorder (MDD) also suffer from at least one comorbid substance use disorder [5]. These teenagers with both major depression and substance use disorders are significantly more likely to suffer from more pronounced psychosocial dysfunction, greater academic problems, more suicidal behavior and completed suicides than those youth without a substance use disorder [6-9].

More specifically, reported drug use has been shown to be a predictor of subsequent suicide attempt in adolescents [10,11] with a positive linear relationship between number of drugs abused and likelihood of suicide attempt [12]. Of concern is the fact that Brent et al. [13] found that having a mood disorder and a substance use disorder may put adolescents at a substantial increased risk for suicide. For these reasons, safe and effective treatments for youths who are suffering from comorbid depression and substance use disorders are needed.

Currently, fluoxetine is the only antidepressant that is labeled by the Food and Drug Administration for use in children and adolescents suffering from MDD [14]. Fluoxetine has been shown to be associated with greater reductions in depressive symptomatology than placebo when administered to depressed youths that do not suffer from substance use disorders [15-17].

In adults, fluoxetine appears to be efficacious in the treatment of adults with MDD and comorbid substance use disorders. For example, Cornelius et al. [18] found that adults with depression and an alcohol abuse disorder who were treated with fluoxetine experienced greater depressive symptom amelioration and a decreased consumption of alcohol in comparison to those subjects who received placebo.

Unfortunately, there are little data available regarding the pharmacological treatment of depressed youths with comorbid substance use disorders despite the prevalence and malignancy of this combination of illnesses. A pilot study of sertraline in 10 depressed adolescents with a substance use disorder showed a significant reduction in depressive symptomatology and alcohol consumed in both the sertraline-treated group and the group that received placebo [19]. An open-label study found that fluoxetine was effective in decreasing both depressive symptomatology and alcohol consumption in adolescents with MDD [20]. In addition, open-label treatment with fluoxetine in substance-abusing adolescents with a comorbid conduct disorder diagnosis who had been abstinent for at least four weeks has been reported to decrease depressive symptoms [21]. These preliminary data in adolescents and placebo-controlled efficacy data in adults suggest a possible role for the use of fluoxetine in the treatment of depressed youth with a comorbid substance use disorder. Furthermore, fluoxetine plus cognitive behavioral therapy (CBT) was found to be superior to CBT plus placebo in adolescents with MDD, conduct disorder (CD), and a substance use disorder in a recently published 16-week study [22].

The purpose of this study was to test the acute efficacy of fluoxetine in reducing depressive symptoms, as well as the safety and tolerability of fluoxetine in the treatment of adolescents with a depressive disorder who also suffered from a comorbid substance use disorder. Surprisingly, an adequately-powered acute randomized controlled trial where subjects receive only either an active drug or placebo for a brief period of time has yet to be published in this population of vulnerable youths. Given the current concerns that have been found regarding the possibility of increased suicidality in adolescents treated with antidepressants [23] and the fact that these comorbid patients appear to be at high risk for self-injurious behavior, particular attention was paid to patient safety. More specifically, as hopelessness has been reported to be strongly associated with suicidal ideation [24,25], this specific construct was monitored throughout the trial.

It was hypothesized that fluoxetine would be superior to placebo in the amelioration of depressive symptomatology in this patient population. Another hypothesis was that treatment with fluoxetine would be associated with a favorable safety and tolerability profile. As the improvement of adolescents with cannabis abuse disorders may be the result of general treatment factors [26], the use of a placebo arm in this trial was considered scientifically rational and ethically justifiable decision. It should also be noted that when this study was designed, published acute pharmacotherapy trials of depressed youths had not included a CBT component. In addition, it was our impression that access to CBT may be limited in community settings. For these two reasons, CBT was not included in this trial's design.

## Methods

All procedures in this study were approved by the University Hospitals Case Medical Center's Institutional Review Board for Human Investigation. Written informed consent was obtained from the subject's guardian and written assent was obtained from the subject prior to any study-related procedures being performed.

### Study design

This was a single site, 8-week, double-blind, placebo-controlled study with 2 parallel arms. Eligible youths were randomized in approximately equal numbers to receive either placebo or fluoxetine during the course of this study. The pre-treatment screening assessments were completed over two visits, generally one week apart. After receiving blinded study medication at the baseline visit, youths returned for assessments after 4 days, and at the end of weeks 1, 2, 3, 4, 6, and 8.

### Subjects

Youths were referred for possible participation from outpatients seen at a clinical research center (CRC) located within an urban, university-based, division of child and adolescent psychiatry; were respondents to advertising; or outpatients seen by other mental health providers from the region.

Outpatients aged 12–17 years, diagnosed with either a current major depressive disorder (MDD) or other depressive disorder that were also suffering from a comorbid substance-related disorder were eligible to enroll into this trial. In addition, eligible patients had to suffer from depressive symptoms of at least moderate severity (Children's Depression Rating Scale-Revised (CDRS-R) total score ≥ 40) [27]. Additional inclusion and exclusion criteria are shown in Table 1.

**Table 1:** Inclusion and exclusion criteria

Youths were included in the study only if they met all of the following criteria:
Male and female outpatients.
Youths whose parent/guardian provided signed informed consent.
Youths who provided signed informed assent.
Youths whose parent/guardian agreed to administer study medication daily.
Youths diagnosed with either a current major depressive disorder or depressive disorder and a comorbid substance-related disorder.
Youths suffering from depressive symptoms of at least moderate severity (CDRS-R score ≥ 40).

Youths were excluded from the study for any of the following reasons:

Youths with a clinically-significant general medical or neurological condition.
Youths with clinical evidence to suggest the presence of mental retardation.
Youths for whom treatment with another psychotropic medication would be anticipated while enrolled in the study.
Youths who received treatment with another psychotropic medication within 2 weeks of receiving blinded study medication.
Youths with a history of intolerance, allergy, or non-response to fluoxetine.
Youths who failed 4 weeks of treatment with a non-TCA, non MAOI antidepressant during the current depressive episode.
Youths with a clinically significant abnormal screening laboratory.
Youths who were actively suicidal, or if in the investigator's judgment participation in the study could place the youth at undue risk.
Diagnosis of any of the following DSM-IV defined disorders: bipolar I or II disorder, psychotic disorder (history), obsessive-compulsive disorder (current), panic disorder (current), bulimia (current), or anorexia (current).
Females who were pregnant or breastfeeding.
Females who were sexually active and were not using medically accepted means of contraception.
Youths who, in the investigator's opinion, required pharmacological detoxification.

#### Subject diagnosis

Current DSM-IV diagnoses were determined based on the results of Schedule for Affective Disorders and Schizophrenia for School-Age Children-Present and Lifetime-Version (KSADS-PL) [28] interview. The KSADS-PL assessment was administered by a child and adolescent psychiatrist or by highly trained research assistants. All research assistants were trained to reach an overall kappa equal to or greater than 0.85 at the item severity level. In addition, diagnoses were confirmed by a clinical evaluation by a child and adolescent psychiatrist. Specification of abuse disorder (abuse vs. dependence), age of onset of symptoms, and length of illness were based on data obtained from the KSADS-PL

### Medication treatment

#### Study medication

Patients were randomized to receive either 10 mg of fluoxetine or matching placebo for the first four weeks of treatment. After four weeks, the dose could be increased to a maximum dose of 20 mg of fluoxetine or matching placebo, based upon the treating physician's discretion.

#### Concomitant medications and therapy

Treatments with other psychotropic medications were not allowed to be prescribed to the subjects during study enrollment. While in the study, youths and their families who were not currently receiving psychotherapy were offered referrals to community-based resources for substance use disorders (Alcoholics Anonymous, etc). Patients and family attendance at such meetings were recorded. If youths were receiving chemical dependency treatment prior to randomization, youths were permitted to continue these psychosocial interventions while in this study. However, patients were excluded if there was a significant increase in the intensity of a community-based psychosocial intervention(s) 2 weeks prior to baseline.

#### Medication adherence

Parents were asked to directly administer the fluoxetine to the teenagers each morning, rather than have the study medication self-administered. Adherence to study medication was assessed by direct inquiry of the parents/patient, by dosing diaries that were to be returned at each study visit, and by pill count calculation.

### Outcome measures

Data were collected by clinicians and research assistants who were blinded to treatment group assignment. The Children's Depression Rating Scale-Revised (CDRS-R) [27] and the Clinical Global Impressions Scale- Severity and Improvement (CGI) [29] were obtained at baseline and the week 1, 2, 3, 4, 6, and 8 study visits. The Beck Depression Inventory (BDI) [30], the Beck Hopelessness Scale (BHS) [31], and the Children's Global Assessment Scale (CGAS) [32] were collected at baseline and week 8 or last week of study participation.

The CDRS-R, a 17-item scale, assesses the severity and presence of depressive symptoms in children and adolescents. Total scores range from 17 to 113, with a score of 40 usually being indicative of clinical depression [27,33]. The CGI severity and improvement scales assess severity and improvements of overall psychiatric illness throughout the duration of the study. CGI Severity (CGI-S) items are rated from 1 (normal, not ill) to 7 (very, severely ill). Furthermore, symptom improvement items on the CGI Improvement (CGI-I) scale are rated from 1 (very much improved) to 7 (very much worse).

Patient-report of depressive symptomatology was assessed with the BDI [30]. The 21 items are summed to obtain a total score that can range from 0 to 63, with scores of 20 or more indicative of moderate to severe depression. The BHS is a valid and reliable measure designed to assess three major areas of hopelessness: feelings about the future, loss of motivation, and expectations [31,34]. The BHS is composed of 20 true-false questions: nine questions are keyed false and 11 are keyed true, with one point being assigned to negative expectations and zero points being assigned to positive expectations. The responses are summed to total scores ranging from 0 to 20. Higher scores are indicative of more hopelessness [35]. Finally, the CGAS [32] is a clinician-completed rating scale that provides a single score that reflects a child or adolescent's overall functional capacity at home, school, and with peers. Scores range from 1 (indicating a severely impaired child) to 100 (indicating a child with superior functioning).

### Safety assessments

#### Substance use monitoring

In order to monitor for substance use, urine toxicology screens were obtained at the screening visit, baseline visit, and at weeks 2, 4, 8, or at the end of study participation. Serum ethanol levels were obtained during the screening process, at the baseline visit and at the end of study. In addition, a research nurse obtained random urine screens at weeks 3 or 6 of the study. Urine screens tested for the presence of amphetamines, opiates, cannabinoids, cocaine, and phencyclidine. Also, at the study physician's discretion, a blood ethanol screen was also able to be obtained at additional study visits if there was a concern about ongoing ethanol abuse. Youth were considered to have had a positive substance use screen if either ethanol and/or any use of the five drugs were detected in blood or urine samples. At each visit, youths were queried about between-visit substance use activity.

#### Safety parameters

Prior to and at each visit during double-blind treatment, resting blood pressure and pulse were measured. In addition, weight was obtained at baseline, week 4, and end of study.

Before patients received study medication, a chemistry profile, hematology profile, thyroid stimulating hormone level, and a urinalysis were obtained. In addition, an electrocardiogram was conducted prior to the patient being prescribed study drug. With the exception of the thyroid stimulating hormone level, all of these laboratories and the electrocardiogram were repeated at the end of study participation. All females had a urine pregnancy test performed at screening and at week 8 or at termination of study participation.

#### Adverse event monitoring

Adverse event data were collected by direct query of the guardians and patients, using the Dosage Record Treatment Emergent Symptom Scale (DOTES) [36] and the Treatment Emergent Symptoms Scale-Write-In (TESS) [37]. Any other adverse events that were spontaneously reported by the patient or guardian were also recorded.

### Statistical analyses

#### Sample size determination

Because no double-blind, placebo-controlled studies of the efficacy of fluoxetine in pediatric patients with depression and comorbid substance use disorders had been conducted at the time this study was designed, the fixed sample size estimate that was employed was based on comparable work in adults. A trial of analogous methodological design in which 25 adult patients were randomly assigned to fluoxetine and 26 to placebo [18] found that fluoxetine was superior to placebo for the treatment of comorbid major depressive disorder and alcohol dependence. In consideration of the results reported by Cornelius et al. [18], the original intention of this trial was to have 30 participants randomized to each treatment arm in order to employ a similarly sized study cohort. However, this study did not have adequate power to detect a medium or small effect size as seen in recent antidepressant trials in youths [38,39].

#### Interim analyses

Due to concerns raised about treatment with fluoxetine and the development of suicidality in youths prescribed this agent [23] and in order to assess whether the risk of exposure to fluoxetine to study participants was justified, a single interim analysis was conducted after approximately 50% of patients per treatment group had completed their participation in the study. Preserving an overall two-sided Type 1 error rate of .05, Lan-DeMets [40] group sequential methods with the most conservative alpha spending function (O'Brien-Fleming) were used to reject the null hypothesis (efficacy boundary, if large treatment differences appear before the end of the study). Whereas repeated testing requires a larger sample size than the fixed sample size counterparts, one look at the data maintains a fixed sample size estimate given the inflation factor is 1.0 [41]. Using results from the interim analysis, a conditional power (CP) computation for futility was also conducted, using guidelines adapted from Lan and Wittes [42]. It was pre-specified that the study would be stopped for futility at t = 0.5 if CP_D(0.5) _<= 0.3.

#### Randomization procedures

Permuted block randomization methods were used to assure a high degree of balance over time and to provide adequate non-predictability [39]. Assignment to fluoxetine or placebo was evenly allocated (1:1 treatment allocation). Randomization was stratified by drug of choice of the youth (either marijuana or alcohol) and current psychotherapy status (either outpatient, other, or no psychotherapy treatment). The randomization sequence was performed using the pseudo-random number generated by SAS, version 6.2 (the SAS Institute, Carey, NC).

The Fisher exact test was used to compare rates of study discontinuation, comorbid psychiatric conditions, concomitant psychotherapy, pre-randomization positive drug toxicology, and dose increases.

#### Efficacy and other psychometric measure analyses

As in many recent studies of juvenile depression, the primary measure used to assess the efficacy of fluoxetine was the CDRS-R [43]. The primary outcome analysis was a random effects mixed model for repeated measurements of CDRS-R scores compared between treatment groups across time. Random-effects estimators included individual patients; fixed-effects estimators included treatment, visit, and treatment by visit interaction using a compound symmetry within-subject variance-covariance matrix. Degrees of freedom for the F test were computed using the Satterthwaite formula, a method that provides a more accurate approximation to the distribution of the F statistic in random effects models than the standard ANOVA method [44]. The baseline and each post-baseline visit were included in the model as the dependent variables.

In addition, a CDRS-R "response" rate was prospectively defined as a ≥ 30% decrease in CDRS-R total score from week 0 to endpoint (last patient visit, weeks 2 to 8). To correct for the nonzero minimum score of the CDRS-R, we used Emslie et al's [16] recommended formula: ([baseline score - 17] - [endpoint score - 17])/baseline score - 17). In addition, patients whose endpoint CDRS-R total score was ≤ 28 were considered "remitted." This definition of remission has been used in other studies of fluoxetine in patients with major depression [16,17].

Time to CDRS-R remission was compared between treatment groups using Kaplan-Meier survival analysis. All other analyses, including mean change in CDRS-R from baseline to endpoint and weekly analyses, were performed on an intention-to-treat basis.

For analysis of CGI-Improvement, only endpoint values were compared, since this scale measures total improvement in direct comparison with patient's condition at baseline. Generalized linear model (GLM) procedures were used for treatment comparisons in continuous change scores in the CDRS-R, BHS, BDI, and CGAS from baseline to endpoint.

#### Toxicology analyses

The post-randomization drug toxicology screens assessed at each study visit were compared across treatment groups using a generalized linear mixed model for discrete outcomes. Using PROC NLMIXED, the initial model included treatment, visit (within-subject factor), pre-randomization drug screen status, and all two-way interactions. Non-significant interactions (p ≥ .10) were dropped from the model.

#### Adverse events

Treatment-emergent adverse events were compared between treatment arms. The Fisher exact test was used to compare the number of occurrences of adverse events rates in the treatment arms. Changes in blood pressure and pulse from baseline to endpoint were compared between fluoxetine and placebo treatment groups using PROC GLM procedures.

Analyses report on data from all patients enrolled who received study medication. All tests of hypotheses were considered statistically significant if the two-sided p value was less than .05. Four additional tests with the CGI-Severity, CGI-Improvement, BHS, and CGAS were conducted. The set of 5 tests were considered to be a family (alternative scales for the same basic outcome of depressive symptoms). Thus the family-wise error rate was set at 0.05. Safety and subgroup analyses were considered secondary. All analyses were performed with SAS software version 9.1.2 (SAS Institute, Cary, NC).

## Results

The trial was stopped after the interim analysis was performed based on the pre-specified futility stopping rule. Comparison of the primary outcome via mixture model analysis crossed the *a priori *futility boundary for early stopping with acceptance of the null hypothesis of no treatment difference in mean change in CDRS-R total score (estimated treatment difference = 0.19, S.E. = 0.58, F = 0.14, p = .74).

An identical conclusion was reached using the comparison between the proportion of patients with a ≥ 30% decrease in CDRS-R total score (fluoxetine: 72%; placebo: 81%: p = .69). It was calculated that if the study had continued to the planned enrollment of 30 patients per treatment arm, the probability of demonstrating a difference in CDRS-R scores between treatment groups was less than 2% under the alternative hypothesis based on the observed treatment group differences.

### Subject demographics

**Figure 1:**
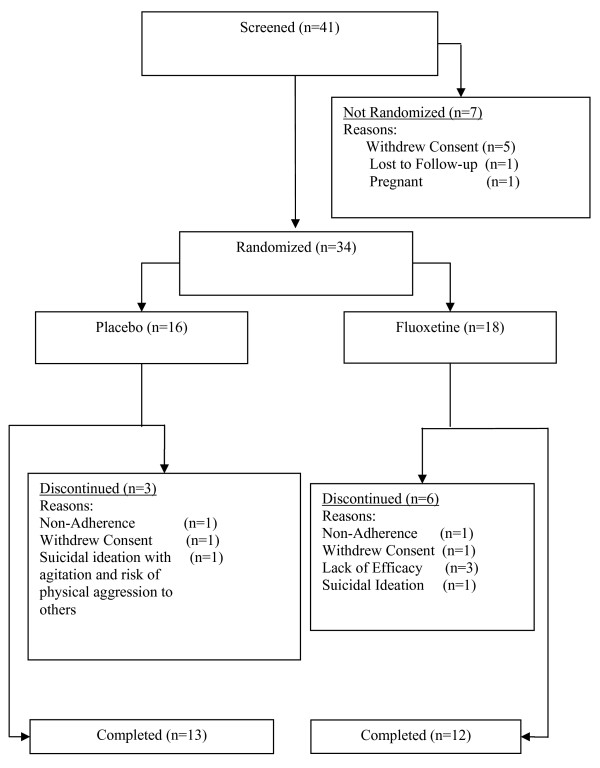
**Patient disposition**.

Eighteen patients were randomly assigned to fluoxetine treatment and 16 to placebo treatment (Figure 1). There were no significant between-group differences in baseline patient demographics (Table 2). Overall, the length of depressive illness ranged between 20–676 weeks and the substance use disorder length ranged from 26–312 weeks. Retrospectively, 26 patients reported that their depressive symptoms occurred first; 6 subjects reported the depression and substance use disorder started simultaneously; one subject reported the onset of the substance use disorder prior to the depressive symptoms; and in one subject this information was not available. Both treatment groups were balanced in the temporal onset of depression and substance use disorders (Fisher's Exact test, p = 1.0).

**Table 2:** Patient demographics

Patient characteristic	Total	Fluoxetine	Placebo	P-value*
	N = 34 (100%)	N = 18 (53%)	N = 16 (47%)	
**Ethnicity, n (%)**				.75

White	25 (73%)	14 (78%)	11 (19%)	
African American	6 (18%)	3 (17%)	3 (69%)	
Other	3 (9%)	1 (5%)	2 (12%)	

**Age (years), mean ± SD**	16.46 ± 1.08	16.55 ± 1.11	16.35 ± 1.08	.61

**Gender, n (%)**				.18

Female	5 (15%)	4 (22%)	1 (6%)	
Male	29 (85%)	14 (78%)	15 (94%)	

**Current Depressive Disorder**				

MDD^a^	29 (85.3%)	15 (83.3%)	14 (87.5%)	1.0
Other Depressive Disorder^b^	5 (14.7%)	3 (16.7%)	2 (12.5%)	.56
Age at onset of depression, mean ± SD	11.41 ± 2.5	11.9 ± 2.7	10.8 ± 2.1	.19
Length of depression, mean ± SD (weeks)	213.1 ± 153.6	185.0 ± 168.3	244.7 ± 133.4	.26

**Current comorbid condition, n (%)**				

ADHD^c^	11 (32.4%)	6 (33%)	5 (31.3%)	1.0
Post Traumatic Stress Disorder	2 (5.9%)	1 (5.6%)	1 (6.3%)	1.0
Conduct Disorder	2 (5.9%)	0 (0%)	2 (12.5%)	.21

**Current SUD**^**d,e**^				

Alcohol	13 (38.2%)	7 (38.9%)	6 (37.5)	1.0
Alcohol Abuse	10 (29.4%)	5 (27.8%)	5 (31.25%)	1.0
Alcohol Dependence	3 (8.8%)	2 (11.1%)	1 (6.25%)	
Cannabis	30 (88.2%)	15 (83.3%)	15 (93.8%)	.60
Cannabis Abuse	16 (47%)	9 (50.0%)	7 (43.8%)	.72
Cannabis Dependence	14 (41.2%)	6 (33.3%)	8 (50.0%)	
Polysubstance	1 (2.9%)	0 (0%)	1 (6.3%)	.47

**Age at onset of SUD^**e**^, mean ± SD**	13.7 ± 1.2	13.8 ± 1.3	13.7 ± 1.3	.86

**Length of SUD, mean ± SD (weeks)**	117.4 ± 68.7	116.0 ± 72.4	118.9 ± 66.8	.91

^a^Major Depressive Disorder; 2 patients with MDD had a comorbid Dysthymic disorder diagnosis

^b^4 patients were diagnosed with Dysthymic Disorder, 3 patients had Depression Not Otherwise Specified

^c^Attention Deficit/Hyperactivity Disorder

^d^Substance Use Disorder

^e^10 patients had more than one substance use disorder

*Fisher's exact test analyses were used to compare percentages; generalized linear model (GLM) procedures were used for comparisons of continuous variables between treatment groups

Of the 34 randomized patients (16 placebo, 18 fluoxetine), 25 completed the 8 weeks of treatment (13 placebo, 12 fluoxetine). Two patients experienced adverse events causing them to discontinue their participation in the study: one (6%) fluoxetine-treated patient discontinued due to being hospitalized for suicidal ideation after three weeks of treatment with 10 mg/day, and 1 (6%) placebo-treated patient discontinued after one week of study participation due to the need for hospitalization for suicidal ideation with agitation and risk of physical aggression to others.

There was no significant difference between treatment groups in the proportion of discontinued patients (p = .45). Treatment groups were equally balanced for discontinuation due to nonadherence with protocol-related procedures (fluoxetine: 1 patient [6%]; placebo: 1 patient [6%]), and patient decision (fluoxetine: 1 patient [6%]; placebo: 1 patient [6%]). Three fluoxetine-treated patients (17%) and no placebo-treated patient discontinued because of ineffective treatment (p = .24).

No treatment group differences were found in participation in psychosocial treatments pre-randomization (fluoxetine 22% versus placebo 6%; p = .34) or post-randomization (fluoxetine 6% versus placebo 19%; p = 0.32). Four fluoxetine-treated patients (25%) and 4 placebo-treated patients (22%) were exposed to psychosocial treatments either at baseline and/or during the trial (p = .85).

### Dosing

Ten of the 15 subjects who were enrolled for a minimum of 4 weeks and were randomized to receive fluoxetine had their dose increased to the maximum dose of 20 mg during the course of the study. All 14 subjects who were randomized to placebo and completed 4 weeks of treatment had their "dose" of blinded medication "increased" to 20 mg. The rate of patients who had their dose increased differed significantly between the two treatment groups (p = 0.042).

### Efficacy assessments

**Figure 2:**
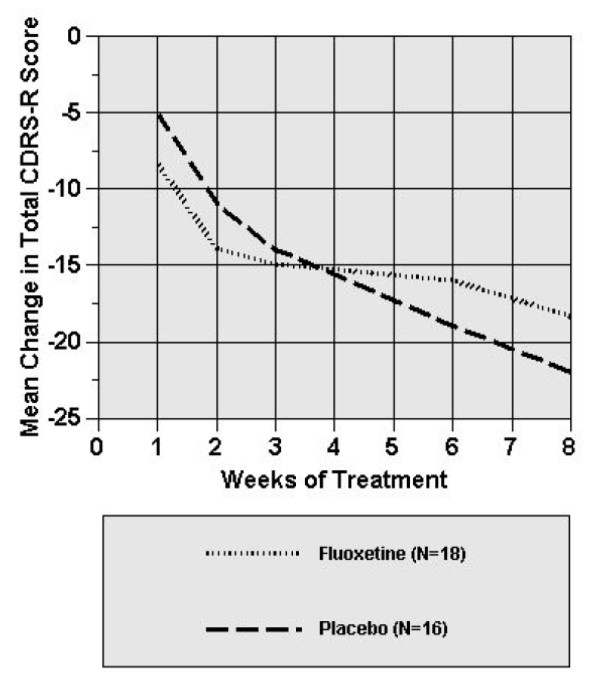
**Mean change from baseline for fluoxetine- and placebo-treated patients on the Children's Depression Rating Scale-Revised**. *Random effects regression model indicated that there was no significant treatment by visit interaction (p = .14).

Placebo-treated patients experienced a greater mean reduction in CDRS-R score than fluoxetine-treated patients at end of study participation (-4.23, 95% confidence interval [CI], -12.95 to 4.49). Although both groups had a reduction in CDRS-R scores, compared with placebo, fluoxetine treatment in this trial was not associated with greater improvement in CDRS-R. More specifically, in the random effects mixture model for repeated measurements, there was no significant treatment by visit interaction (p = .14), indicating no difference between treatment groups in mean change in CDRS-R score at any week during the trial. As shown in Figure 2, those subjects that received placebo showed a greater decrease in CDRS-R scores from baseline beginning at week 5 in comparison to those subjects that received fluoxetine. Using recommended methods [45], primary outcome results remained constant when the extreme CDRS-R score observed (most severe/least severe) was assumed for these 3 fluoxetine-treated patients.

Fifty percent of patients in both treatment groups met the *a priori *defined criteria for remission (p = 1.0). Furthermore, using survival analyses, there were no significant differences between treatment groups in time to CDRS-R remission (CDRS-R ≤ 28; Wilcoxon X2 = 0.5, df = 1, p = 0.83).

#### Other psychometric analyses

Table 3 includes mean psychometric scores at baseline and end of study. Half of all fluoxetine-treated patients (50%) were rated much or very much improved (CGI-Improvement score of 1 or 2) compared with 38% of placebo-treated patients (p = 0.51). As can be seen in Table 3, no significant differences between treatment groups in improvement were found in CGI-severity score, BHS score, BDI score, and CGAS score. The finding that the 95% confidence interval of the difference between treatment groups in mean change in CGI-Improvement, CGI-Severity, BHS, BDI, and CGAS scores each span zero confirms that the two treatment groups did not separate significantly on mean improvements on any scale utilized in this clinical trial.

**Table 3:** Change in baseline to endpoint in depressive symptomatology and psychosocial functioning

Measure characteristic	Baseline	Fluoxetine^a ^Endpoint	Change	Baseline	Placebo^b ^Endpoint	Change	Difference in Change^c^ (95% CI)	p^d^	F
CDRS-R	53.0 ± 2.32	34.60 ± 3.22	-18.40 ± 2.94	53.94 ± 2.46	31.31 ± 3.42	-22.63 ± 3.12	-4.23 (-12.95–4.49)	.33	0.98

CGI-S	4.28 ± 0.15	2.88 ± 0.28	-1.39 ± 0.28	4.37 ± 0.16	2.87 ± 0.30	-1.50 ± 0.30	-0.11 (-0.95–0.73)	.71	0.07

CGI-I	--	2.61 ± 0.32	--	--	2.44 ± 0.34	--	-0.17 (-1.12–0.77)	.79	0.14

BDI	17.20 ± 3.10	7.62 ± 1.96	-9.58 ± 3.29	13.00 ± 3.33	8.12 ± 2.11	-4.88 ± 3.54	4.70 (-5.23–14.63)	.34	0.95

BHS	7.33 ± 1.38	4.07 ± 1.10	-3.27 ± 1.38	7.69 ± 1.49	5.46 ± 1.18	-2.23 ± 1.48	1.04 (-3.12–5.19)	.61	0.26

CGAS	53.06 ± 2.06	69.63 ± 3.62	16.56 ± 3.50	51.21 ± 2.20	65.93 ± 3.87	14.71 ± 3.75	1.85 (-8.67–12.37)	.72	0.13

Values represent mean ± SE from fixed effects parameter estimates. CDRS-R = Children's Depression Rating Scale-Revised; CGI-S = Clinical Global Impressions – Severity scale; CGI-I = Clinical Global Impressions – Improvement Scale; BDI = Beck Depression Inventory; BHS=Beck Hopelessness Scale; CGAS = Children's Global Assessment of Functioning

^a^Fluoxetine: last observation carried forward: for BDI, n = 15, for BHS, n = 15, for CGAS, n = 16.

^b^Placebo: last observation carried forward: for BDI, n = 13, for BHS, n = 13, for CGAS, n = 14.

^c^Difference in Change shows the difference in average change score of fluoxetine-treated patients in comparison to placebo-treated patients. The 95% confidence intervals for the differences are shown in parentheses below. If the entire 95% confidence interval is greater than zero, this indicates a 95% or greater probability that the mean change associated with fluoxetine treatment is greater than the mean change associated with placebo.

^d^Generalized linear model (GLM) procedures were used for analysis of continuous baseline and endpoint values; Value for difference in mean change between treatment groups, using Type III difference of least square means.

#### Drug toxicology screen analyses

**Figure 3:**
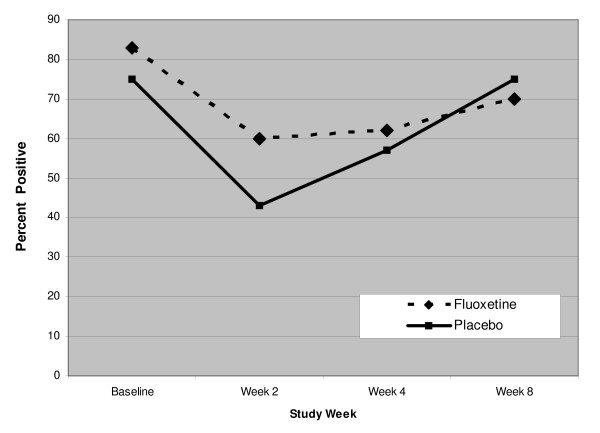
**Percent of patients with positive drug tests who received fluoxetine or placebo for 8 weeks**.

Fourteen of the 16 of subjects in the placebo group and 13/18 subjects in the fluoxetine group had random urine toxicology tests obtained at either week 3, week 6, or both time points. No between treatment group differences were found in pre-randomization rates of positive drug toxicology (fluoxetine: 83% versus placebo 75%; p = .68). Post-randomization rates of any positive drug toxicology at each study visit are presented in Figure 3. As shown in Figure 3, there was no significant difference in rates of positive drug toxicology between treatment groups at any post- randomization visit (F = 0.22, df = 1, p = 0.65). In addition, over the course of the trial, no alcohol was detected in the serum ethanol levels.

### Safety assessments

No statistically significant between-group differences in treatment emergent adverse events were observed during the 8 weeks of this study (Table 4).

**Table 4:** Side effects ratings by treatment group

Symptom	Total 34 (100%)	Fluoxetine 18 (53%)	Placebo 16 (47%)	P*
Headache	18 (53%)	10 (56%)	8 (50%)	0.75
Nasal Congestion	13 (38%)	7 (39%)	6 (38%)	0.67
Drowsiness	8 (24%)	6 (33%)	2 (13%)	0.97
Nausea/Vomiting	8 (24%)	5 (28%)	3 (19%)	0.85
Stomach Pain	7 (21%)	2 (11%)	5 (31%)	0.21
Diarrhea	4 (12%)	2 (11%)	2 (13%)	0.65
Dry Mouth	3 (9%)	1 (6%)	2 (13%)	0.45
Syncope/Dizziness	3 (9%)	2 (11%)	1 (6%)	0.86
Insomnia	3 (9%)	1 (6%)	2 (13%)	0.45

*Fisher exact test was used to compare occurrences of adverse events across treatment groups.

In addition, there were no statistically significant differences in changes from baseline and end of treatment in systolic blood pressure, diastolic blood pressure, and pulse between the treatment groups (all p values > .05). Furthermore, no statistically significant differences for changes from baseline and end of study in weight were found between treatment groups. In addition, there were no clinically significant blood pressure, pulse, electrocardiogram, or laboratory assessments noted during the course of the trial.

## Discussion

In this study, fluoxetine was found not to be superior to placebo in the acute treatment of depressive symptoms in depressed adolescents with a concomitant substance-related disorder. In addition, those patients treated with fluoxetine did not show a significantly greater decrease in their substance use in comparison to those patients who received placebo. However, it should be noted that the assessment of substance use described in this report was based on the number of positive drug screens rather than actual quantities of substances used. Therefore, it is possible that the amount of drug use significantly diminished but the frequency of use remained the same.

A key observation was the high placebo response rate seen in this trial. The baseline characteristics of these youths suggest that they were substantively symptomatic prior to receiving treatment under the auspices of this clinical trial. In prior acute treatment studies of MDD in youths without substance use disorders, fluoxetine was found to be superior to placebo [15-17]. In those trials, youths had baseline mean CDRS-R scores ranging from approximately 55 to 61 in the treatment groups. It is possible that the more modest depression severity of this cohort contributed to the high placebo response rate. Whether or not the presence of a substance use disorder influences placebo response is a question worthy of further study.

Although fluoxetine was not found to be superior to placebo on the primary outcome measure, the percentage of subjects in both treatment groups who were rated much or very much improved (CGI-Improvement score of 1 or 2), appears to be similar to rates of response that have been found in previous fluoxetine treatment studies of depression in youths free of substance use disorders [15-17]. The discrepancy between CDRS-R score reductions and CGI-I response rates may have occurred as a result of the fact that response based on CGI-I ratings reflect overall improvement of clinical status in a comorbid population whereas the CDRS-R solely considers depressive symptomatology.

In a randomized controlled trial of fluoxetine and CBT in adolescents with major depression, behavior problems, and substance use disorders, greater efficacy in combined fluoxetine and CBT treatment in comparison to placebo and CBT treatment emerged subsequent to 8 weeks of study participation into a trial period of 16 weeks [22]. Therefore, the length of the trial described herein, as well as absence of conjunctive CBT, may have affected our ability to detect a difference between the two treatment groups.

Owing to concerns regarding tolerability in a group of youths who were at risk for ongoing substance abuse, the dose of fluoxetine used in this study was limited to a maximum of 20 mg/day. Although the efficacy of higher doses of fluoxetine was not examined, it should be noted that other studies [16,17] found a significant difference between youth treated with 20 mg of fluoxetine in comparison to subjects who received placebo. Furthermore, when considering the substantive response rate seen in this trial, it appears that despite the use of a comparatively limited dose range of fluoxetine, patients were not under-dosed in this study.

Caution should be taken when interpreting the results of this study. A key limitation of this study is its small sample size and resulting low statistical power. Furthermore, the brevity of this study prevents definitive conclusions about the safety of fluoxetine treatment in this study population from being made. Additionally, because this study was designed to be 8 weeks in length, like other placebo-controlled fluoxetine trials in youths [16,17], whether or not separation from placebo may have become evident with a longer treatment duration remains an empiric question.

As noted above, another main limitation is the high placebo response rate. Other methodological shortcomings include: 1) the serum ethanol measurements employed in this study designed were ineffective in detecting alcohol consumption; and 2) the degree of marijuana abuse was not quantified as part of the drug screens used in this trial. It should be noted that serum ethanol screens were used because of issues pertaining to feasibility. There also were concerns that more exhaustive testing for substances of abuse would have likely increased subject burden to a degree that it would have adversely influenced recruitment.

Another potential limitation is that this study did not specifically recruit youths suffering with only one specific substance abuse disorder. Also, despite the fact that youths with other types of substance-related disorders might have been eligible for study participation, the patients studied in this trial primarily suffered from alcohol and/or marijuana use disorders.

As mentioned earlier, owing to concerns regarding fluoxetine tolerability in this particular group of adolescents, the dose of fluoxetine used in this study was limited to 20 mg/day. Therefore, it is not clear whether or not the study subjects could have derived more substantive benefits from higher doses of fluoxetine.

In addition, this study did not exclude youth who met criteria for depression disorders other than MDD. Although less than 15% of youths had depression disorders other than MDD, a low proportion of the sample well-balanced across treatment groups at baseline, it is possible that this wider range of subject diagnoses may have influenced this trial's results. However, despite its limitations, this study demonstrated the feasibility of doing such a trial in this challenging population.

## Conclusion

In this study, short-term treatment with fluoxetine was not superior to placebo in alleviating depressive symptoms or decreasing rates of positive drug screens in adolescents with depression and a substance use disorder. However, fluoxetine was found to be reasonably well-tolerated when compared to placebo. Further research, specifically secondary analyses of the study outcomes, may help provide insights into why these findings diverge from results from previously conducted studies in which fluoxetine was shown to be superior to placebo in youths with and without substance use disorders.

## Competing interests

RLF receives or has received research support, acted as a consultant and/or served on a speaker's bureau for Abbott, Addrenex, AstraZeneca, Bristol-Myers Squibb, Forest, GlaxoSmithKline, Johnson & Johnson, Lilly, Neuropharm, Novartis, Organon, Otsuka, Pfizer, Sanofi-Aventis, Sepracore, Shire, Solvay, Supernus Pharmaceuticals, and Wyeth. MDR has received research grant support, acted as a consultant or served on a speaker's bureau for Abbott Laboratories, Astellas, AstraZeneca, Bayer, Bristol-Myers Squibb, Diiachi-Sankyo, Eli Lilly, Enzon, Forrest Laboratories, GlaxoSmithKline, HRSA, Janssen, Johnson & Johnson, Merck, NICHD, Novartis, Organon, Pfizer, Roche, Sanofi Aventis, Schering, Somerset, State of Ohio-Department of Health, UCB Pharma, Wyeth-Ayerst. The other authors have no financial ties to disclose.

## Authors' contributions

All authors have made substantial contribution to the conception, design, and/or conduct of the study, have been involved in the drafting and/or critical revising of this manuscript, and all authors have given final approval of this manuscript.
